# Differential responses to maternal diabetes in embryo and visceral yolk sac

**DOI:** 10.3389/fcell.2023.1273641

**Published:** 2023-10-19

**Authors:** J. Michael Salbaum, Kirsten P. Stone, Claudia Kruger, Claudia Kappen

**Affiliations:** ^1^ Department of Regulation of Gene Expression, Pennington Biomedical Research Center, Louisiana State University System, Baton Rouge, LA, United States; ^2^ Department of Developmental Biology, Pennington Biomedical Research Center, Louisiana State University System, Baton Rouge, LA, United States

**Keywords:** diabetic pregnancy, neural tube defect, somite number, somite stage, RNA-seq, visceral yolk sac, non-obese diabetic mouse strain

## Abstract

**Introduction:** Maternal diabetes during pregnancy is well known to be associated with a higher risk for structural birth defects in the offspring. Recent searches for underlying mechanisms have largely focused on aberrant processes in the embryo itself, although prior research in rodent models implicated dysfunction also of the visceral yolk sac. The objective of our research was to investigate both tissues within the conceptus simultaneously.

**Methods:** We conducted unbiased transcriptome profiling by RNA sequencing on pairs of individual yolk sacs and their cognate embryos, using the non-obese diabetic (NOD) mouse model. The analysis was performed at gestational day 8.5 on morphologically normal specimen to circumvent confounding by defective development.

**Results:** Even with large sample numbers (*n* = 33 in each group), we observed considerable variability of gene expression, primarily driven by exposure to maternal diabetes, and secondarily by developmental stage of the embryo. Only a moderate number of genes changed expression in the yolk sac, while in the embryo, the exposure distinctly influenced the relationship of gene expression levels to developmental progression, revealing a possible role for altered cell cycle regulation in the response. Also affected in embryos under diabetic conditions were genes involved in cholesterol biosynthesis and NAD metabolism pathways.

**Discussion:** Exposure to maternal diabetes during gastrulation changes transcriptomic profiles in embryos to a substantially greater effect than in the corresponding yolk sacs, indicating that despite yolk sac being of embryonic origin, different mechanisms control transcriptional activity in these tissues. The effects of maternal diabetes on expression of many genes that are correlated with developmental progression (i.e. somite stage) highlight the importance of considering developmental maturity in the interpretation of transcriptomic data. Our analyses identified cholesterol biosynthesis and NAD metabolism as novel pathways not previously implicated in diabetic pregnancies. Both NAD and cholesterol availability affect a wide variety of cellular signaling processes, and can be modulated by diet, implying that prevention of adverse outcomes from diabetic pregnancies may require broad interventions, particularly in the early stages of pregnancy.

## Introduction

Maternal diabetes is a risk factor for structural birth defects, most prominently heart defects and neural tube defects (Martinez-Frias, 1994). Molecular mechanisms potentially underlying these defects have been uncovered in the past 2 decades, largely on the basis of rodent models (Gabbay-Benziv et al., 2015; Ornoy et al., 2015). Multiple reports have implicated reduced expression of specific transcription factors, oxidative and hypoxic stress, apoptotic and altered signaling pathways, as well as involvement of microRNAs and ER stress (Phelan et al., 1997; Yang et al., 1997; Li et al., 2005; Wentzel and Eriksson, 2005; Wang et al., 2015; Ramya et al., 2017; Wang et al., 2017). However, many of these studies were performed with focus on particular molecules, and even the few untargeted surveys published (Jiang et al., 2008; Sato et al., 2008; Pavlinkova et al., 2009) were conducted at or after mid-gestation, when embryonic defects were already manifest.

Using a genetic model, the non-obese diabetic (NOD) mouse strain that faithfully recapitulates metabolic and immunological characteristics of human Type I diabetes (Leiter, 2001), we previously demonstrated that migration of embryonic mesodermal cells from the primitive streak is impaired in diabetic pregnancies, leading to cell accumulation in the midline of the neural plate and formation of protrusions that physically hinder closure of the neural tube (Salbaum et al., 2015; 2022). These studies identified cellular alterations in diabetic conditions that precede formation and closure of the neural tube.

Furthermore, based upon culture of rat and mouse conceptuses, prior work proposed that compromised yolk sac function would deprive the embryo of critical nutrients, such as arachidonic acid (Pinter et al., 1986a) and prostaglandin (Piddington et al., 1996; Wentzel and Eriksson, 2005), leading to the “yolk sac hypothesis of diabetic embryopathy” (Reece et al., 1994). Our own recent work demonstrated excess accumulation of lipids in visceral yolk sac in two independent mouse models of diabetic pregnancy (Zhang et al., 2023), indicating altered nutrient availability to the embryo from the yolk sac at time points as early as E7.5 and E8.5. However, comprehensive gene expression profiles from yolk sac at such early stages, under conditions of maternal diabetes, have not been reported to date.

Finally, diabetic pregnancies present with incomplete penetrance of defects that -dependent on the particular model-occur at most in half of the developing embryos (Ornoy et al., 2015) while the majority of embryos develop normally and are viable after birth. This resiliency may be explained by interactions between the yolk sac and the embryo within each conceptional unit, which remain critically under-investigated. We therefore here conducted unbiased genome-wide transcriptome profiling of embryos and their cognate yolk sacs at day E8.5 of development, comprising the earliest stages of neural tube closure.

## Materials and methods

### Animals

Animal husbandry as well as all procedures were performed as described previously (Dobin et al., 2013; Salbaum et al., 2015; 2022), with approval of the Institutional Animal Care and Use Committee of Pennington Biomedical Research Center. Briefly, mice of the strain NOD/ShiLtJ (Strain #: 001976) were obtained from The Jackson Laboratory (Bar Harbor, ME). To check for the onset of diabetes, blood glucose was monitored weekly using a commercial glucometer. Once female mice exceeded a blood glucose level of 250 mg/dl, they were deemed diabetic and mated to normoglycemic males of the same strain. Female mice of the same cohort that had normal blood glucose levels were used as controls. The day of appearance of a vaginal plug after mating was designated day 0.5 of gestation. Dissections of embryos were done at 8.5 days of gestation; maternal blood glucose level was recorded before euthanasia. Females were euthanized using controlled CO_2_ inhalation, secondary euthanasia was done via heart puncture, and decidua were dissected from the uterus in phosphate-buffered saline (PBS). Decidua were transferred to OPTI-MEM medium (Thermo Fisher) supplemented with 5% fetal bovine serum (Thermo Fisher), and embryos were dissected free from decidual material using a Leica M16 stereomicroscope. Ectoplacental cone and parietal yolk sac were removed. The visceral yolk sac was dissected and transferred into Trizol for RNA extraction. Embryos were inspected for stage-appropriate morphology; embryos that displayed malformations were not used in the study. The number of somite pairs was recorded, and embryos were transferred into Trizol for RNA extraction.

### RNA extraction, library preparation, and RNA sequencing

RNA was extracted using the Trizol protocol (Kappen et al., 2022), and quality was assessed on an Agilent Bioanalyzer 2,100 (Agilent Technologies, Santa Clara, CA); only samples with an RNA Integrity Index larger than 7 were used in subsequent experiments. 50 ng of total RNA per sample were used to construct Illumina-compatible RNA sequencing libraries, applying the Lexogen 3′-Quant-Seq method, and utilizing Unique Molecular Identifiers (UMI) in order to guard against PCR-based artefacts when counting sequencing reads (reagents and protocol from Lexogen, Inc., Greenland, NH). After passing quality control performed on an Agilent Bioanalyzer 2,100, barcoded libraries for all samples were pooled and sequenced together (single ended, 75 bases) on an Illumina NextSeq 550 instrument (Illumina, Inc., San Diego, CA).

### Bioinformatics and data analysis

Sequencing data were obtained in the form of fastq files. After adapter trimming, sequences were aligned to the mm10 reference using STAR software (Dobin et al., 2013). Duplicate reads originating from the PCR amplification step in the library preparation protocol were eliminated using the collapse_UMI_BAM software utility (Lexogen, Inc.); consequently, each UMI-corrected read represents one mRNA molecule, and the sum of reads per gene reflects the expression level of that gene, respectively. Gene hit counts were obtained using HTSeq software (Putri et al., 2022), resulting in a raw count table for all embryo and all visceral yolk sac samples. The average number of UMI-corrected 3′-end reads per sequencing library was 2.4 M. After removal of genes with less than three read counts per sample, we obtained 13,425 ENSEMBL gene IDs for embryo samples, and 12,442 ENSEMBL gene IDs for yolk sac samples for use in subsequent analyses. The data have been uploaded to the Gene Expression Omnibus database (GSE197396).

Sex of each sample was inferred after RNA sequencing by examining the expression of specific genes located on either the X or the Y chromosome. High expression of the X chromosome gene Xist, together with concomitant absence of expression of the Y chromosome genes Ddx3y, Uty, and Kdm5d, was used to designate a sample (embryo and corresponding yolk sac) as female. In contrast, lack of Xist expression, together with expression of Ddx3y, Uty, and Kdm5d, was taken to identify a sample as male.

Embryo and visceral yolk sac sequencing data were analyzed separately. Differential gene expression between samples from diabetic vs. normal pregnancies was assessed using DESeq2 software (Love et al., 2014). Correction for multiple testing was done via the Benjamini-Hochberg procedure implemented in DESeq2, and genes with an adjusted *p*-value below 0.1 were considered to be statistically significantly different between metabolic conditions. Hierarchical clustering was performed in Cluster3 (de Hoon et al., 2004) and visualization was done in TreeView, both available as Open Source Software from http://bonsai.hgc.jp/∼mdehoon/software/cluster/software.htm#ctv.

To identify genes where expression levels were related to developmental age of the conceptus (using the number of embryo somite pairs as proxy), we calculated the Pearson coefficient for the correlation of each gene to somite number. For more detailed analyses to identify the influence of age and sex on gene expression patterns, raw count data were normalized and scaled, using procedures implemented in Seurat software (Hao et al., 2021). Subsets of the scaled data set -differentially expressed genes as defined by DESeq2 analysis and somite stage-correlated genes (with an absolute Pearson Correlation coefficient larger than 0.5)- were the basis for principal component analyses (PCA) in the statistical computing environment R, using the software packages FactoMiner (Lê et al., 2008), factoextra [http://www.sthda.com/english/rpkgs/factoextra], as well as the utility sjmisc (Lüdecke, 2018). Plots were generated using ggplot2 (Wickham, 2016) https://ggplot2.tidyverse.org.

For differential gene expression driven by maternal diabetes, as well as for somite stage-correlated gene sets, gene pathway analyses were performed using Ingenuity Pathway analysis software (Qiagen, Redwood City, CA) with default settings; *p*-values in all analyses were adjusted for multiple testing by the Benjamini-Hochberg algorithm. Annotations with Z-scores smaller than |1|, as well as annotations lacking unequivocal prediction of activation or inhibition, were not considered. All analyses were current to the IPA platform as of 31 July 2023.

## Results

**FIGURE 1:**
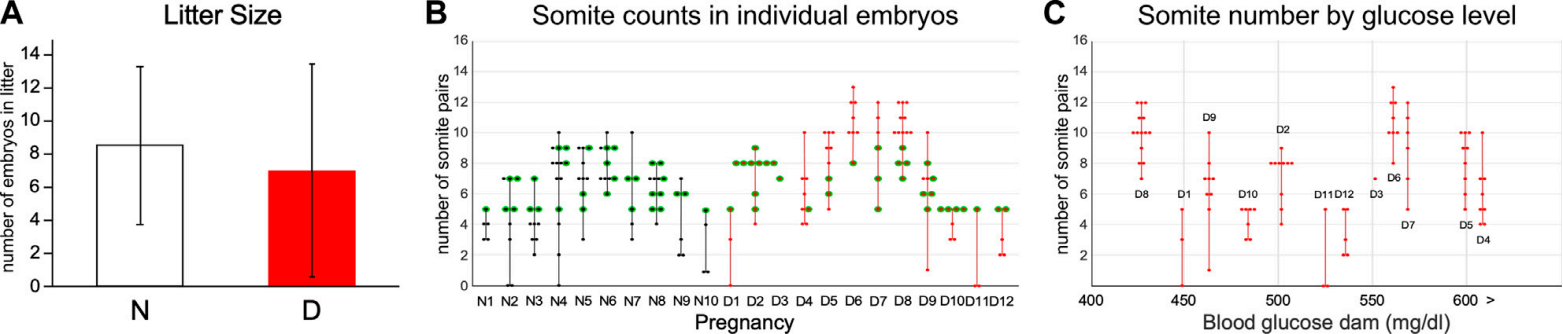
Characteristics of Pregnancies: Litter size and somite counts. This study comprised samples from 10 normoglycemic pregnancies and 12 diabetic pregnancies. **(A)** Litter sizes were not significantly different between metabolic conditions. **(B)** Somite counts of individual embryos recovered from each pregnancy. While the overall spread of somite counts was larger across all diabetic pregnancies, there was no significant difference in the spread of somite stages/individual pregnancy when normal and diabetic conditions were compared. Only embryos with between 5 and 9 somite pairs (green outlines) were included in the transcriptome analyses, with selection for inclusion in the study depending on RNA amounts recovered and RNA quality, as well as quality of the sequencing library. **(C)** Somite counts and litter size are plotted relative to the blood glucose level of pregnant diabetic dams at the time of euthanasia. There was no correlation of either somite number/embryo (coefficient R^2^ = 0.0006), spread of somite counts within a pregnancy (R^2^ = 0.0078, not shown) or litter size (R^2^ = 0.06, not shown) to maternal blood glucose level in diabetic pregnancies.

In this study, we set out to obtain unbiased genome-wide transcriptional profiles of conceptuses from mouse pregnancies affected by maternal diabetes. Implantation sites were collected at 8.5 days of gestation from normoglycemic and diabetic pregnancies in the non-obese diabetic (NOD) strain of mice, and embryos and their associated visceral yolk sacs were dissected. Blood glucose levels for the dams in both experimental groups are listed in Table 1, with additional sample characteristics given in Table 2. Litter sizes were not significantly different between both metabolic conditions (Figure 1A). Somite counts of embryos within each litter are depicted in Figure 1B; there was no apparent correlation of maternal blood glucose level with litter size or somite number (Figure 1C). Embryos with a number of somite pairs between 5 and 9 were included in RNA-Seq studies (samples with green outline in Figure 1B).

**TABLE 1:** Pregnancy characteristics.

Pregnancy	Blood glucose in dam		# of embryos
#	At mating	At dissection	
N1	102	108	5
N2	109	119	9
N3	99	135	9
N4	112	118	14
N5	86	126	10
N6	97	109	10
N7	103	122	6
N8	93	125	11
N9	87	105	6
N10	89	164	4
			
D1	312	449	3
D2	253	506	10
D3	267	553	1
D4	458	>600	8
D5	433	595	9
D6	410	563	8
D7	255	568	6
D8	418	423	14
D9	368	461	9
D10	>600	478	7
D11	293	524	3
D12	448	538	5

**TABLE 2:** Characteristics of experimental samples.

Condition	Embryos	Yolk sacs
Normal	33	33
Diabetic	33	33
Total	66	66
		
Female	29	29
Male	37	37
Total	66	66
		
Somite Pairs	5	24
	6	10
	7	11
	8	13
	9	8
Total		66

### Exposure to maternal diabetes elicits differential transcriptomic responses in embryos and yolk sacs

**FIGURE 2:**
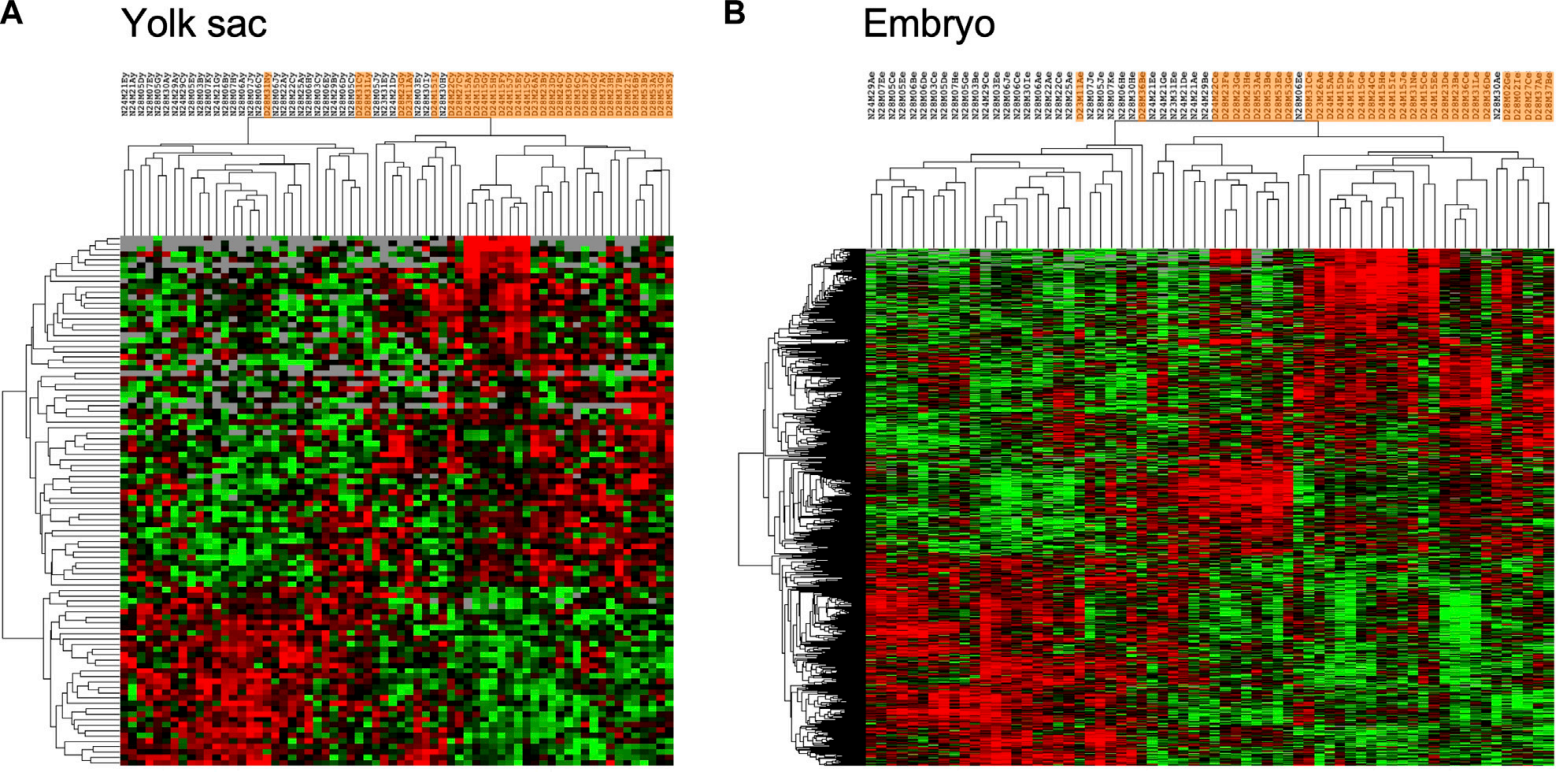
Genes with significantly different expression in yolk sacs and embryos from normal and diabetic pregnancies. Raw sequence data were processed as described in the Methods section, and normalized read counts based upon Unique Molecular Identifiers (UMI) were subjected to comparisons with DESeq2 software; comparisons with adjusted *p*-value smaller than 0.1 were considered indicative of differential gene expression. Hierarchical clustering was performed in Cluster 3 separately for yolk sac and embryo samples, and visualized as heat maps in TreeView, respectively. Red color intensity represents the extent of fold-change increase in diabetes-exposed relative to normal samples, and green color intensity depicts the fold-change decrease; grey indicates non-significant fold-change in a given sample. Orange shading identifies data from diabetes-exposed specimen. **(A)** 98 IDs passed the significance criteria in comparisons including 33 yolk sac samples for each metabolic condition. **(B)** 891 IDs passed the significance criteria in comparisons of embryo samples.

Comparisons of expression profiles obtained for samples from normal to those from diabetic pregnancies were performed using DESeq2 software, and are shown in Figure 2, in the form of heat maps. For yolk sac samples, a moderate number of 98 IDs passed the threshold of significance (adjusted *p*-value of 0.1, Figure 2A), with 65 IDs expressed at higher levels and 33 IDs at lower levels in samples from diabetic when compared to normal pregnancies (Supplementary File S1). The magnitude of changes exceeded 1.5-fold for 45 IDs with increased and for 5 IDs with decreased expression. Cluster analyses reveal incomplete resolution between experimental groups, represented by the intermingling of samples from diabetic pregnancies (highlighted in orange) with normal samples in several areas of the heat map. The low number of differentially expressed genes indicates that visceral yolk sacs do not undergo major transcriptome changes in response to the exposure to maternal diabetes.

In contrast, a much higher number of 891 genes showed significantly altered expression in embryos from diabetic dams compared to normal pregnancies (Figure 2B), with 528 genes exhibiting increased (203 by more than 1.5-fold), and 363 genes exhibiting decreased (29 by more than 1.5-fold) levels. The hierarchical clustering algorithm achieves better separation of experimental groups, likely due to the larger number of differentially expressed genes (Supplementary File S2). Canonical Pathways detected as enriched within this gene set by IPA (Table 3) include cholesterol biosynthesis, which is predicted to be reduced in the diabetic condition. Predicted to be activated are the GDP-mannose/Colanic Acid biosynthesis pathways, as well as components involved in NAD metabolism and signaling. Taken together, the RNA-seq results show that effects of maternal diabetes are evident at the transcriptional level at least as early as E8.5, before and during formation of the major organ systems in the embryo, and that the yolk sac is not a prominent site of transcriptional response to exposure.

**TABLE 3:** IPA of genes with differential expression in embryo upon exposure to maternal diabetes.

Ingenuity canonical pathways	-log (*p*-value)	Ratio	z-score
Superpathway of Cholesterol Biosynthesis	6.90	0.30	−2.530
Ferroptosis Signaling Pathway	3.18	0.12	−1.941
Mevalonate Pathway I	2.55	0.36	−1.000
NAD Signaling Pathway	2.06	0.09	2.138
GDP-mannose Biosynthesis	1.67	0.50	n.d
Colanic Acid Building Blocks Biosynthesis	1.62	0.29	2.000
ATM Signaling	1.58	0.10	1.134

### Factors contributing to variability of gene expression

**FIGURE 3:**
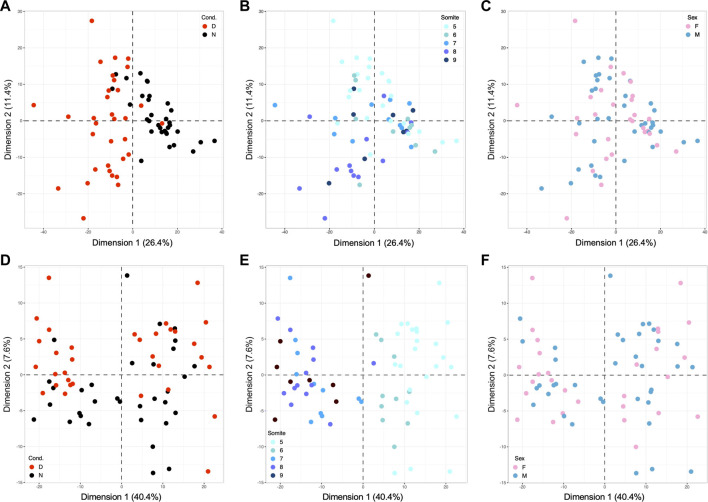
Principal Component Analyses identify metabolic condition and developmental stage as major drivers of gene expression in E8.5 embryos. PCA was performed using the R computing platform and the FactoMiner software module. **(A)** Based upon 891 differentially expressed genes, the contribution of the strongest component, Dimension 1, accounts for 26.4% of the overall variation; coloring of samples by known metabolic state of the originating pregnancy identifies the exposure to maternal diabetes as explanatory factor for Dimension 1. **(B)** The same PCA plot colored for the number of somite pairs in each embryo from 5 to 9, with smaller numbers represented in lighter, and larger numbers in darker color. This graph identifies developmental stage as Dimension 2, contributing 11.4% of the variation. **(C)** Coloring for embryonic sex did not reveal any influence of the two components shown, nor of 8 additional dimensions tested (up to Dimension 10, data not shown). **(D)** PCA analysis based upon 460 genes with strong Pearson correlation to somite stage, colored for metabolic condition. This diagram identifies the exposure to maternal diabetes as discriminating factor along Dimension 2, explaining 7.6% of the variation among clustering based upon somite stage. **(E)** The same PCA plot colored for somite state displays strong separation of samples long Dimension 1, which explains 40.4% of the results driven by the factor developmental age. **(F)** Coloring for embryonic sex again did not reveal any influence in up to 10 Dimensions (data not shown).

However, the heat maps also reveal that expression patterns are not uniform within experimental groups, with seemingly greater variation among the diabetes-exposed samples than among samples from normal pregnancies. To quantify inter-individual variation in gene expression profiles and to identify possible causes, we performed Principal Component Analysis (PCA) on subsets of genes, in order to detect relevant factors (components) explaining portions of the variation, and to attribute their weight relative to each other and the overall outcome. Figure 3A depicts PCA results for the 891 genes differentially expressed in embryonic samples (the dataset represented in Figure 2B), with red color overlay marking exposure to diabetic conditions. As expected, the analysis reveals samples from normal and diabetic pregnancies largely separated along Dimension 1. These results show that the largest extent of expression variation in this gene set (26.4%) is attributable to the exposure to maternal diabetes.

Yet, the spread of samples along Dimension 2 is considerable, prompting us to ask whether this component could reflect developmental maturity. We therefore marked the individual datapoints in this analysis with colors indicating the number of somite pairs in each embryo (Figure 3B). This graph showed that samples generally segregate according to their developmental stage along Dimension 2. Although there is some overlap between somite stages, it is noteworthy that embryos from normal pregnancies (largely on the right side of the graph) are distributed in a narrower range along Dimension 2 (from −11 to +13), when compared to embryos from diabetic pregnancies (largely on the left side, with a range from −27 to +27.5). We therefore conclude that Dimension 2, developmental stage, is the second major source of variation in the gene expression profiles, particularly prominent in diabetes-exposed individuals.

The potential influence of sex of the embryo was examined by overlaying information on embryonic sex onto the same PCA plot underlying Panels A and B, as shown in Figure 3C. This reveals that samples are not separated by sex along Dimensions 1 and 2. We also did not find separation by sex along any of the next lesser dimensions (examined up to Dimension 10; data not shown). This finding is not in itself surprising when expression of genes linked to sex chromosomes is considered, as they account for a minor fraction of differentially expressed genes in this dataset: only 30 IDs map to the X-chromosome. But even beyond expression of sex-chromosome-encoded genes, the response to maternal diabetes is not appreciably different between embryos of male and female sex. Taken together, these PCA analyses -based upon differentially expressed genes- uncover exposure to maternal diabetes, in combination with developmental stage, as the major factors impacting gene expression in embryos from normal and diabetic pregnancies.

### Role of developmental stage in transcriptomic responses in the exposure to maternal diabetes

The strong contribution of developmental progression motivated us to ask whether correlations to particular genes can be identified. We calculated the Pearson coefficient for correlation of expression level of each gene with somite stage. We selected those genes with values between 0.5 and +1 as strongly correlated, and genes between −1 and −0.5 as strongly anti-correlated (for details see Figure 5) and conducted separate Principal Component Analysis on this subset of somite-stage-correlated genes. The PCA results clearly ascertains somite stage as the major factor along Dimension 1 (Figure 3E), accounting for 40.4% of the variation in this gene set; expectedly so, as they were selected for correlation with somite number. Coloring for metabolic condition reveals Dimension 2 as explaining 7.6% of the variation (Figure 3D), with more diabetes-exposed samples in the positive (upper) area of the graph (*n* = 23) than in the negative (lower) space (*n* = 10), whereas normal samples are preferentially classified into the lower (*n* = 24) than upper area (*n* = 9) (*p* = 0.0012 in a 2-sided Fisher’s exact test). These data demonstrate the interaction of diabetes-exposure with developmental progression, even within the group of genes solely selected on the basis of correlation with somite stage. Lastly, we also examined the role of sex as a component in Pearson-correlated genes (Figure 3F). As before, we did not detect clustering of samples by sex (along any of the first 10 dimensions); here, 21 out of 459 IDs with location information map to the X-chromosome (4.57%). Taken together, these data illustrate the extent of variation contributed by developmental maturity of each individual embryo, and emphasize the need to take developmental stage into consideration when interpreting gene expression profiles.

**FIGURE 4:**
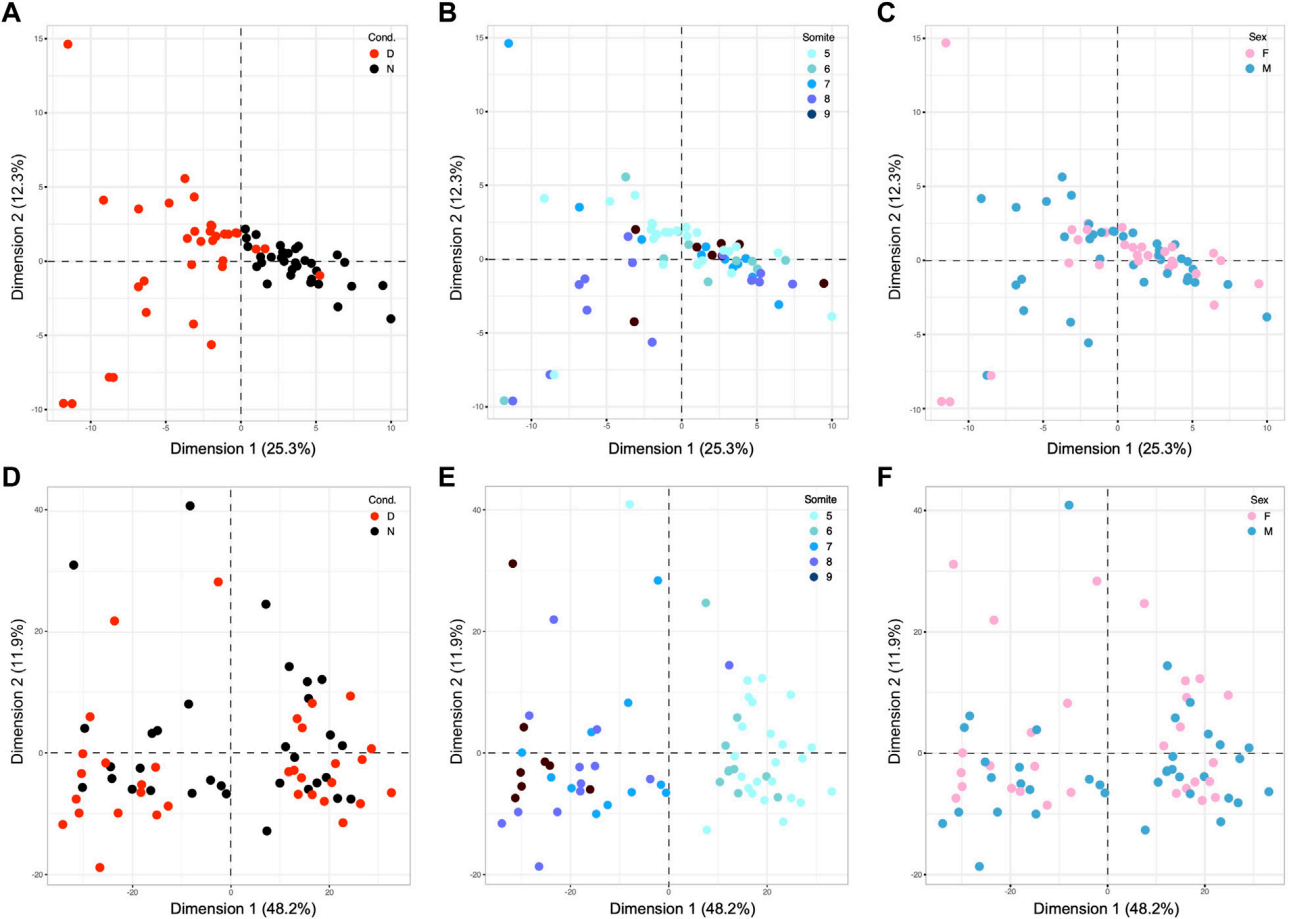
Principal Component Analyses of results for visceral yolk sac samples from E8.5 embryos. **(A–C)**: PCA for yolk sac samples based upon 98 differentially expressed genes, with coloring scheme analogous to Figure 2. **(A)** The exposure to maternal diabetes is revealed as the major component that accounts for 25.3% of overall variation **(B)** The distribution of samples along Dimension 2, which contributes 12.3% of the variation, only moderately discriminates samples based upon somite stage, likely because of the very small number of underlying genes. **(C)** Sex of the samples was not a discriminating factor in the two dimensions shown, nor any other tested (up to Dimension 10, data not shown). Panels **(D,E)**: PCR based upon 980 genes with strong Pearson coefficients for correlation with the number of somite pairs in the cognate embryo for each yolk sac sample. **(D)** Although more diabetes-exposed samples appear in the negative space of Dimension 2, the difference to the number of normal samples in the same area was not significant. **(E)** Coloring of the same diagram for somite stage reveals discrimination of samples along Dimension 1, highlighting developmental stage as the major explanatory factor in this dataset. **(F)** As before, sex of the cognate embryo was not a discriminating factor along all 10 dimensions tested (data not shown).

We then applied the same analysis workflow to yolk sac-expressed genes. Principal Component Analysis of genes that are differentially expressed when samples from normal and diabetic pregnancies are compared (Figure 4A) identifies maternal diabetes as the largest factor (Dimension 1) explaining 25.3% of the variation between yolk sac gene expression profiles from normal and diabetic pregnancies. Once again, Dimension 2, which accounts for 12.3% of the variation, contributes to a much larger spread among diabetes-exposed samples largely on the left (from −9.5 to +14.5) when compared to spread between samples from normal (from −4 to +2.4) pregnancies on the right of the graph. Thus, yolk sac gene expression profiles recapitulate our earlier finding for embryo profiles. Coloring for somite stage indicates rather moderate separation along Dimension 2 (Figure 4B), likely due to the small number of only 98 differentially expressed genes available for this analysis (of which only 3 map to the X-chromosome, 3.1%). As before, sex of the sample (Figure 4C) was not revealed as a driving factor, even when up to the tenth dimension was examined (data not shown). Thus, exposure to maternal diabetes and developmental stage of the corresponding embryo are the major factors contributing to the gene expression profiles for yolk sac samples.

Principal Component Analysis based upon Pearson correlation coefficients for yolk sac-expressed genes also yielded results comparable to our findings for embryos: the cognate embryo’s developmental stage, whether correlated or anti-correlated, accounted for almost half of the variation (48.2%) in yolk sac gene expression profiles (Figure 4E). Exposure to maternal diabetes (Figure 4D) was a lesser factor, and sex (Figure 4F) had negligible explanatory power (tested up to Dimension 10, data not shown) in this set of somite-stage-related genes (of which 39 IDs map to the X-chromosome = 4%). As before, these results highlight the strong influence of developmental stage on the gene expression profiles in yolk sacs, with even more prominent effect in diabetes-exposed specimen.

### Influence of the exposure to maternal diabetes on gene expression related to developmental age

**FIGURE 5:**
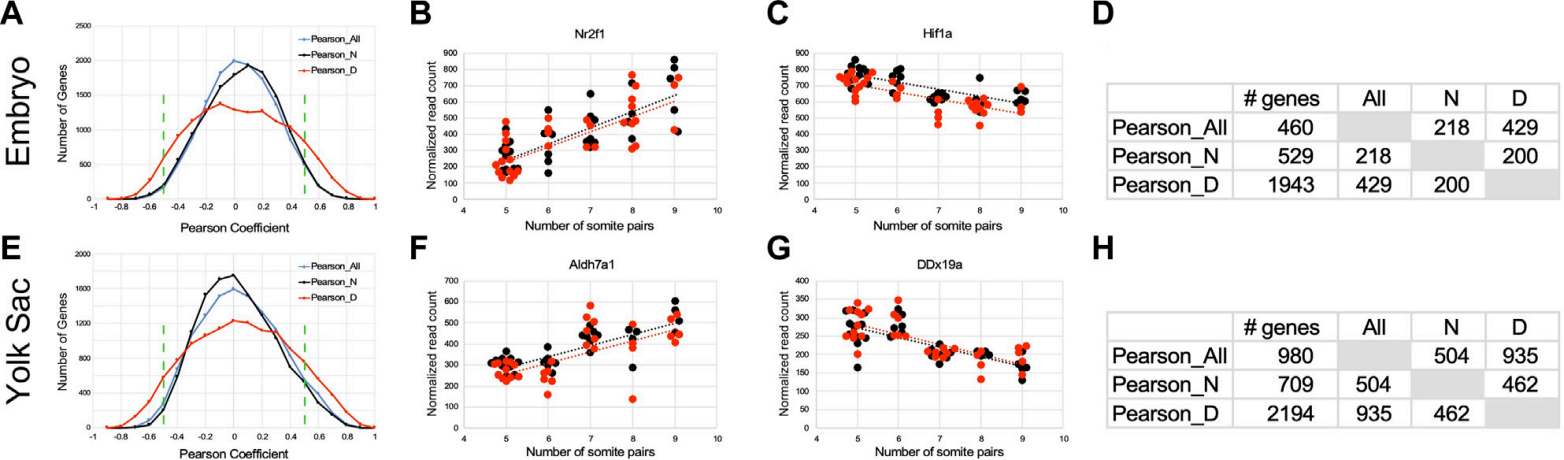
Interaction of exposure to maternal diabetes with developmental stage-related gene expression. Pearson correlation coefficients for each gene relative to the number of somite pairs were calculated based on all 66 samples (Pearson_All), and for the 33 samples from normal pregnancies (Pearson_N) and from the 33 samples from diabetic pregnancies (Pearson_D) separately. **(A–D)**: Results for Embryo samples. **(A)** The histogram for gene expressed in embryos shows the distribution of Pearson coefficients from −1 to +1, with anti-correlations on the left side, and positive correlations on the right side of the graph. Green stippled lines show the cutoff for selection as strongly anti-correlated (from −1 to −0.5) and strongly correlated (from +0.5 to +1) genes. Note the flatter and wider distribution of coefficient values in diabetes-exposed samples, which results in a larger number of genes fulfilling the cut-off criteria. Panel **(B)** Example of a gene (Nr2f1: encoding transcription factor COUP-TF1) strongly correlated to somite stage (dotted lines; black: normal condition, red: diabetes condition). **(C)** Example of a gene strongly anti-correlated to somite stage (Hif1a: encoding hypoxia-inducible factor 1 alpha), again with stronger correlation in diabetes-exposed samples. **(D)** The table for embryo samples also displays a larger number of genes with coefficients fulfilling the selection criteria in diabetes-exposed samples, highlighting that well over 1,000 genes are uniquely correlated/anti-correlated to developmental stage in embryos exposed to conditions of maternal diabetes during pregnancy. Panels **(E–H)**: Analogous results for Yolk Sac samples. **(E)** The distribution of Pearson coefficients for yolk sac samples also displays flatter and wider distribution of coefficient values in diabetes-exposed samples, which results in a larger number of genes fulfilling the cut-off criteria. **(F)** Example of a gene (Aldh7a1: aldehyde dehydrogenase family 7, member A1) strongly correlated to somite stage. **(G)** Example of a gene strongly anti-correlated to somite stage (DDx19a: DEAD-box helicase 19a). **(H)** The table for yolk sac samples also displays a larger number of genes with coefficients fulfilling the selection criteria in diabetes-exposed samples, again highlighting a unique effect of maternal diabetes on developmental progression in yolk sac samples.

Next, we sought to identify particular genes whose expression is correlated, or anti-correlated, with somite stage. Because somite stage (Dimension 2 in Figure 3A) contributed to expression variation to a greater extent in diabetes-exposed embryos, we also calculated Pearson coefficient values separately for both experimental groups. Coefficients calculated across all 66 individuals in the study exhibited the distribution depicted in the blue line in Figure 5A. Limiting the calculation solely to individuals from normal pregnancies yielded a curve with very similar shape (black line). Intriguingly, the curve for correlations for individuals from diabetic pregnancies (red line) appears flatter, with greater contribution of values to the tails of the distribution. The larger number of genes correlated with developmental stage in embryos from diabetic pregnancies compared to normal pregnancies indicates a distinct effect of maternal diabetes on gene expression during developmental progression.

An example of correlation with increasing number of somites is shown in Figure 5B; an example of anti-correlation, with gene expression levels decreasing with developmental progression is shown in Figure 5C. Using the cut-off criteria as described, we identified 529 genes in normal embryos where expression was positively correlated (252 IDs) or anti-correlated (277 IDs) to somite stage, and in embryos from diabetic pregnancies, a total of 1943 genes were correlated (990 IDs) or anti-correlated (953 IDs) to somite stage. Only half of those genes were identical between normal and diabetic pregnancies (Figure 5D), with a larger number uniquely related to somite stage in the diabetic condition. These results substantiate our earlier finding that maternal diabetes distinctly alters gene expression it relates to developmental age of the embryo.

Analogous results were obtained with yolk sac data: the overall distributions of Pearson coefficients identify a larger number of genes correlated with somite stage of the cognate embryo in diabetes-exposed samples than under normal conditions (Figure 5E). 469 genes were positively correlated (example in Figure 5F) and 240 genes were anti-correlated (example in Figure 5G) to developmental age in yolk sacs from normal conditions, and in yolk sacs from diabetic conditions 1,167 genes were positively correlated and 1,027 genes anti-correlated. Again, only about half of those genes were identical between diabetes-exposed and normal yolk sacs, with a larger number uniquely affected by diabetes exposure (Figure 5H), just as in the corresponding embryos. Taken together, these results for yolk sacs, as well as their corresponding embryos, highlight the interaction of diabetes exposure with developmental progression in yolks sac and embryo.

**FIGURE 6:**
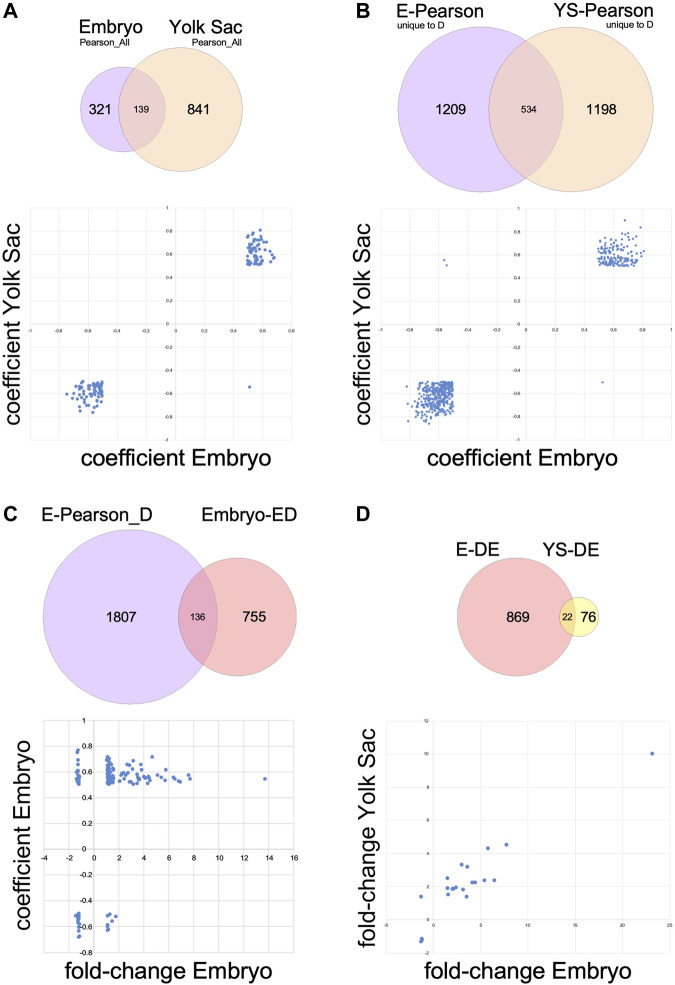
Relationships of transcriptome changes in corresponding embryo and yolk sac tissues. Underlying datasets used in the comparisons are shown in Venn diagrams; the plots in the lower half of each panel feature data represented in the overlap of the circles. **(A)** Genes expressed in both embryo and yolk sac that are correlated or anti-correlated with somite stage according to Pearson correlations calculated over all 66 samples in each tissue (Pearson_All). In 138 out of 139 cases, the relationship of expression level to somite stage is congruent between embryo and yolk sac. **(B)** Genes expressed in both embryo and yolk sac that are somite stage-correlated only in diabetes exposure conditions (genes also correlated in samples from normal conditions were removed from these datasets). For all but three genes out of 534, the relationship of expression level to somite stage is congruent between embryo and yolk sac. **(C)** Genes with differential expression in the comparison of embryos exposed to maternal diabetes to normal embryos that are also uniquely affected by maternal diabetes in their correlation to somite stage. As expected, this dataset contains genes that are positively correlated or anti-correlated (separated along the Y-axis), with increased or decreased expression levels in diabetes-exposed embryos (X-axis). **(D)** Genes with differential expression upon diabetes exposure that are expressed in both embryos and yolk sacs. Generally, the direction of change after exposure to maternal diabetes is congruent between yolk sac and cognate embryo, except for one gene (in the lower portion of the upper left quadrant).

Finally, we examined the direction of correlation for each gene in embryo relative to its correlation for yolk sac, in either the dataset calculated on the basis of all samples (Pearson_All; 139 genes in common between embryo and yolk sac), as shown in Figure 6A, or from the genes whose correlation to somite stage was uniquely affected by exposure to maternal diabetes (Pearson_D minus Pearson_N; 534 genes in common between embryo and yolk sac), shown in Figure 6B. In more than 99% of cases, the direction of correlation matched between embryo and yolk sac; the significance of the rare exceptions (1/139 = 0.72% and 3/534 = 0.56% genes) remains to be established.

### Biological pathways related to developmental stage

We then subjected lists of genes correlated or anti-correlated with developmental stage for embryos and yolk sacs, respectively (data in Supplementary Files S3, S4) to Ingenuity Pathway Analyses. The results for embryos are depicted in Table 4. Strongly correlated to increasing somite number are DNA repair (BER) and translation control (EIF2) pathways, and pathways involved in cell adhesion and motility. Representation of genes encoding proteins with roles in mitochondrial function is also prominent, and overlaps with estrogen receptor and neutrophil trap pathways, and oxidative phosphorylation pathways are predicted to be activated. Anti-correlated genes link declining activity of oncogenic (CML, WNK, and HOTAIR) and micro-RNA biogenesis pathways with increasing somite number. The parallel analysis for yolk sac-expressed genes with correlation to somite stage of the cognate embryo (Table 5) also returned pathways involved in control of translation and mitochondrial activity. Anti-correlated genes indicate declining involvement of DNA damage repair and replication control with increasing somite number; a reduction of transcription complex assembly is also predicted. Similar to embryonic cells, micro-RNA biosynthesis and signaling pathway activity appears to be declining, too. One caveat in these analyses is the limited sample number available at each somite stage. Likewise, the criteria for inclusion of genes as significant (Pearson coefficients between |0.5 and 1|) were arbitrarily chosen, and the number of genes in any IPA analysis is well-known to affect pathway annotation results. Notwithstanding, with sufficient sample number, it may be possible in the future to achieve higher resolution of gene expression signatures that are specifically diagnostic for each somite stage.

**TABLE 4:** IPA of Embryo-expressed genes with strong Pearson correlation to somite stage.

positively correlated genes:			
Ingenuity canonical pathways	-log (*p*-value)	Ratio	z-score
Oxidative Phosphorylation	4.42	0.143	4.000
Granzyme A Signaling	3.57	0.160	−3.464
Mitochondrial Dysfunction	3.48	0.078	−3.530
Estrogen Receptor Signaling	3.48	0.073	3.153
EIF2 Signaling	3.48	0.091	3.051
BER (Base Excision Repair) Pathway	1.74	0.159	2.646
Neutrophil Extracellular Trap Signaling Pathway	1.74	0.063	5.000
Regulation of Actin-based Motility by Rho	1.56	0.096	3.317
Remodeling of Epithelial Adherens Junctions	1.47	0.118	2.000
Signaling by Rho Family GTPases	1.47	0.067	4.000

negative correlated genes:			
Ingenuity Canonical Pathways	-log (*p*-value)	Ratio	z-score
Chronic Myeloid Leukemia Signaling	2.16	0.076	−3.710
WNK Renal Signaling Pathway	1.75	0.104	−2.236
HOTAIR Regulatory Pathway	1.51	0.080	−2.496
MicroRNA Biogenesis Signaling Pathway	1.51	0.075	−2.138

Negative correlated (anti-correlated) genes.

**TABLE 5:** IPA of Yolk Sac-expressed genes with strong Pearson correlation to somite stage.

positively correlated genes:			
Ingenuity canonical pathways	-log (*p*-value)	Ratio	z-score
EIF2 Signaling	5.48	0.100	3.357
Oxidative Phosphorylation	4.16	0.125	3.742
Mitochondrial Dysfunction	2.47	0.064	−3.411
Neutrophil Extracellular Trap Signaling Pathway	1.31	0.053	3.273

negative correlated genes:		
Ingenuity Canonical Pathways	-log (*p*-value)	Ratio	z-score
Role of BRCA1 in DNA Damage Response	3.56	0.150	−2.828
MicroRNA Biogenesis Signaling Pathway	2.69	0.086	−3.000
Cell Cycle Control of Chromosomal Replication	2.24	0.143	−2.828
Nucleotide Excision Repair, Enhanced Pathway	2.24	0.111	−2.646
Assembly of RNA Polymerase II Complex	1.92	0.140	−2.646

Negative correlated (anti-correlated) genes.

The importance of considering somite stage becomes further evident during annotation of the effects of maternal diabetes on embryo gene expression: 15.3% of the 891 differentially expressed genes in embryos are contained within the Pearson_D gene set for embryos (Figure 6C), i.e., they belong to genes uniquely affected by the exposure in their correlation to somite stage (Supplementary File S5), and thus strongly contribute to both Dimensions 1 and 2 (in Figure 3A, B). While pathway analyses were precluded due to the limited number of genes (139), more consideration on their functional roles is offered in the Discussion.

Only a few genes were differentially expressed in Yolk Sacs upon exposure to maternal diabetes (Figure 2A). Pathway Analysis did not return any annotations of significance, after correction for multiple testing. However, as shown in Figure 6D, 22 yolk sac-expressed genes were also differentially expressed in the embryo, which allowed us to test whether they were affected to the same extent by the diabetes exposure. Indeed, all genes with increased expression in embryo were also increased in yolk sac in diabetic pregnancies, and decreases in yolk sac were matched by decreased expression levels in yolk sac, except for Six5, where expression was moderately increased by the exposure in yolk sac, and slightly decreased by exposure in embryos, although the embryonic expression level was generally higher.

### Relationship of responses to exposure between embryos and their cognate yolk sacs

**FIGURE 7:**
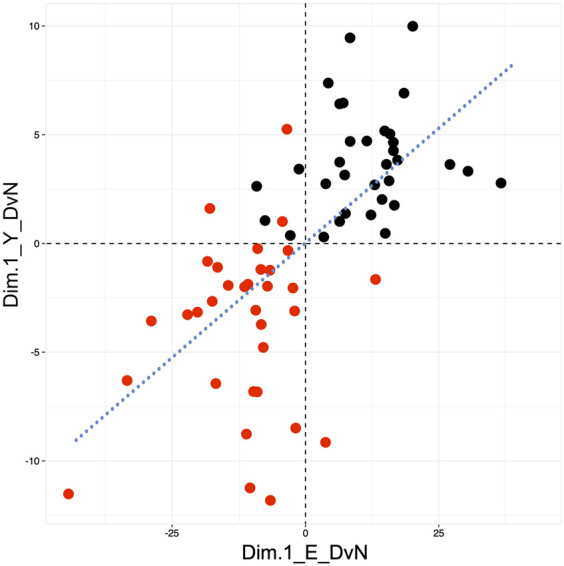
Coordination of the response to diabetes-exposure between yolk sacs and their cognate embryos. Composite scores for yolk sac and embryo genes that are differentially expressed after exposure to maternal diabetes were taken from Dimension 1 (value along the X-axis) of the PCA analyses depicted in Panels A of Figures 3 (for embryo data) and 4 (for yolk sac data), respectively. Individuals from normal pregnancies (black) cluster largely in the upper right quadrant of the graph, while individuals from diabetic pregnancies (red) cluster predominantly in the lower left quadrant. Note the larger scatter for diabetes-exposed individuals. Proximity to the regression line conveys the overall correspondence of changes in yolk sac and embryo from the same individual, as far as captured by the PCA composite score.

Finally, we sought to determine whether the overall transcriptome response to diabetes exposure in yolk sac was correlated to the response in the corresponding embryo, using individual conceptuses as the unit of comparison. As a composite value for each sample, we used its location along Dimension 1 of the PCAs for differentially expressed genes, which discriminates individuals by the major component “diabetes exposure.” When the tissue pair data for each conceptus are plotted, as depicted in Figure 7, the graph reproduces the distribution of values along each axis as in the Dimensions 1 of Panels A in Figures 3 (for embryo) and 4 (for yolk sac), respectively. As before, individuals from diabetic pregnancies are largely clustered in one quadrant, left and lower, and individuals from normal pregnancies in the right and upper quadrant. Proximity to the regression line indicates that the extent of response in both tissues is similar within the same conceptus, whereas deviation from the regression diagonal reflects differences in response magnitude, as well as influence of other explanatory factors on yolk sac and/or cognate embryo. Finding such deviations may suggest that even in normal development, but clearly more so in exposure to diabetes (note the larger scatter between exposed individuals), there could be some extent of “un-coordination” between both tissues of the same conceptus. A caveat here is that the basis for discrimination along the yolk sac axis is only a small number of 89 genes, of which 22 (24.7%) are also expressed in the embryo (Figure 6D). But, in advantage over previous studies, our analyses are based upon 33 individuals in each experimental group, at different somite stages, which -as we showed here-contributes to overall variation in the transcriptome profiles.

## Discussion

In this study, we generated unbiased transcriptome profiles of individual conceptuses from a mouse model of diabetic pregnancy. Our goal was to identify molecular signatures of the exposure to maternal diabetes prior to the manifestation of structural birth defects, such as neural tube defects. Because the visceral yolk sac plays a critical role in supplying nutrients to the embryo before the placenta is fully functional, a second focus in our analyses was on relationships within each embryo-yolk sac unit. A particular strength in our approach is the inclusion of a large number of conceptuses (*n* = 33 each in control and experimental group, respectively), which came from 10 independent normal and 12 independent diabetic pregnancies, respectively. RNA-seq data were obtained separately from individual samples of embryo and cognate yolk sac. For each embryo, we also recorded developmental maturity, by virtue of the number of somite pairs present, and we included only morphologically normal embryos with between 5 and 9 somite pairs in the investigation.

### Identification of novel pathways in the transcriptomic responses of conceptuses to maternal diabetes

By comparing RNA-seq profiles from diabetes-exposed embryos to those from normal pregnancies, our initial analysis identified close to 900 genes whose expression levels were different, after passing criteria for statistical significance and adjustment for multiple comparisons. One of the canonical pathways we identified as deregulated in embryos from diabetic pregnancies is cholesterol biosynthesis, predicted to be reduced by the exposure. Before formation of the placenta, the embryo relies on the maternal circulation for the supply of cholesterol (Woollett et al., 2000; Herman, 2003), which is taken up and processed by the visceral yolk sac. Lipid transporters, including ApoB and SR-B1, are required in this process, as demonstrated by neural tube and other defects in knockout mutants (Farese et al., 1995; Farese et al., 1996; Santander et al., 2013; Santander et al., 2017). Biosynthesis of cholesterol involves a complex series of enzymatic steps that require Acetyl-CoA as the building block (Mitsche et al., 2015), and mutations in many of the involved enzymes result in developmental defects (Farese and Herz, 1998). Reduction of cholesterol biosynthesis in diabetes-exposed embryos would be expected to affect membrane synthesis and fluidity, protein prenylation (Tricarico et al., 2015), and signaling pathways, including hedgehog signaling (Radhakrishnan et al., 2020). While the relevance of these pathways to malformations in diabetic pregnancies remains to be investigated, to our knowledge, this is the first implication of cholesterol deficiency in diabetes-induced birth defects.

Conversely, predicted to be activated is NAD metabolism, which is required in many enzymatic and signaling processes (Feuz et al., 2023), including redox-signaling. NAD levels in pregnancy are dependent on intake of Niacin, and diets low in Niacin were linked to a higher prevalence of birth defects (Palawaththa et al., 2022). Mice with mutations that reduce NAD availability exhibit developmental defects (Cuny et al., 2020; Cuny et al., 2023) and suggest that low NAD levels could be involved in teratogenesis upon exposure to various agents (Mark et al., 2022). Our finding of predicted increased activity of NAD metabolism in diabetes-exposed embryos could be indicative of a compensatory response. Moreover, NAD is also a cofactor for Poly (ADP-ribose) polymerases (Pollard et al., 2022) with roles in transcription and stress responses (Langelier et al., 2018), inhibition of cell proliferation (Tuncel et al., 2012), DNA repair (Morales et al., 2014), and remodeling of chromatin (Hassa and Hottiger, 2008). It is currently unknown whether NAD levels in post-implantation embryos are changed by exposure to maternal diabetes, and which of the underlying mechanisms would be affected, but our results provide a rationale for future investigation.

In addition to clear distinctions based upon maternal metabolic disease, we here noticed considerable variability of expression profiles even among individuals within the same experimental group. Such variability could be related to the extent of maternal hyperglycemia, size of litters, sex of the embryo, or its developmental maturity. Glucose levels at mating and the time of sacrifice of the diabetic females had no effect on litter size when compared to normal pregnancies (data not shown). Even among embryos from normal pregnancies, where maternal glucose level is tightly regulated (Kappen et al., 2011), variability of transcriptome profiles was evident (Figure 2A); thus, the presence of variability is unlikely to be related to glucose levels. Our analyses were also unable to detect a role of embryonic sex in this variability; in addition to the rather small fraction of transcripts derived from genes located on the sex chromosomes, the nonappearance of transcriptome differences by sex may also be related to our matching of female and male embryos for somite stage in the analysis.

### Identification of biological pathways correlated with developmental progression and their modulation by exposure to diabetes

In contrast, the overlay of somite stage information on PCA analyses of differentially expressed genes revealed the influence of developmental maturity of individual embryos on the overall variation of expression repertoires. Notably, embryos from diabetic pregnancies exhibited greater variability compared to normal pregnancies (Figure 3). Furthermore, ranking of genes by correlation of their expression level to the number of somite pairs formally identified groups of genes with changes of expression over time. Intriguingly, the positive correlation of mitochondrial activity and oxidative phosphorylation pathways -in both embryo and yolk sac samples-matches well with reports of the switch from glycolysis towards oxidative phosphorylation in embryonic metabolism in the later developmental stages we investigated here (Johnson et al., 2003; Yamaguchi et al., 2017). Also consistent in this regard is the anti-correlated decline of expression of glycolysis-stimulating Hif1a (Figure 5C) with increasing developmental maturity of the embryo.

We observed a broader distribution of Pearson correlations, in both positive and negative direction, for gene expression in diabetes-exposed compared to normal embryos (Figure 5). The greater prevalence of stronger Pearson correlations demonstrates a unique effect of the diabetes exposure on the relationship of gene expression to developmental stage. To our knowledge, this is the first report of maternal diabetes affecting developmental progression at the molecular level, and the underlying mechanisms remain to be investigated. Also unresolved is whether this response to the exposure affects all cells in the embryo equally, or particular tissues and cell types preferentially.

**FIGURE 8:**
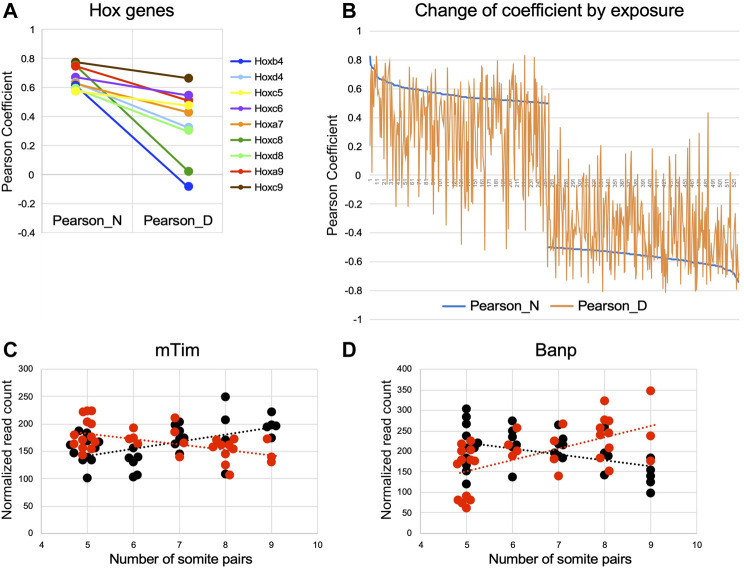
Exposure to maternal diabetes alters the relationship of gene expression level to somite stage Pearson coefficients were plotted for selected genes in a comparison between mormal and diabetes exposed embryo samples. **(A)** Hox genes with correlation to somite number between |1 to 0.5|; exposure to maternal diabetes decreases correlation coefficients for all Hox genes depicted. **(B)** Genes with positive coefficients in the normal condition are plotted on the left half of the graph, genes with anti-correlation to the right side. Coefficient values in the normal condition are given in blue, coefficient values in diabetes exposure conditions are plotted vertically, with deviation from normal indicated in orange. Weakening of correlations (towards coefficients close to 0) is evident for normally correlated and anti-correlated genes; strengthening of correlations is less prevalent. **(C)** Example of a gene, encoding the mouse Timeless homolog, where a normally (black) positive correlation to somite number is turned into negative correlation upon diabetes exposure (red). **(D)** Example of a gene, encoding BTG3-associated nuclear protein Banp, where a normally anti-correlated gene is switched into positive correlation by diabetes exposure.

To further illustrate the effect, we first consider two genes whose correlation to somite stage is equally strong in both experimental groups: Nr2f1 (Figure 5B), encoding a nuclear transcription factor involved in forebrain development, displays positive correlation with increasing somite number in normal (coefficient of 0.734) as well as in diabetes-exposed embryo samples (coefficient of 0.744). Transcript levels for Hif1alpha (Figure 5C), the transcription factor indicative of hypoxic conditions, are anti-correlated in normal embryo samples (coefficient of −0.74) and also in diabetes-exposed embryos (coefficient of −0.712), meaning Hif1a expression levels generally decline with increasing developmental progression of the embryo, irrespective of metabolic condition. In contrast, Hox gene expression levels, which positively correlate with increasing somite number in normal embryos, show weakened correlations in embryos exposed to maternal diabetes (Figure 8A). Moreover, 9 Hox genes are found among the top 100 positively correlated genes in normal conditions, whereas only one Hox gene is represented in the 528 genes with comparable coefficient values in diabetic conditions. We interpret these results as indicative of “un-coupling” of Hox gene expression and developmental maturity by the exposure to maternal diabetes. Indeed, this phenomenon is evident for many genes (Figure 8B; gene list in Supplemental File S6), where a strong correlation found in normal pregnancies is weakened by the exposure to maternal diabetes, or -in rare instances-even switched to an anti-correlation (example in Figure 8C). Similarly, diabetes exposure can reverse a normally negative into a positive correlation (example in Figure 8D), or enhance normally weak correlations (data not shown). Taken together, these results further support our conclusion that maternal diabetes not only increases overall variability in gene expression profiles, but also affects embryonic progression to developmental maturity.

Strong correlations to somite stage that were uniquely detected only in the diabetic condition were enriched for transcripts encoding histones, which are also among the genes differentially expressed when diabetes-exposed and normal embryos are compared (Supplementary File S5). The particular histone transcripts are known to be “replication-dependent” (Suzuki et al., 2023) and are increased during S-phase of the cell cycle (Fritz et al., 2023), annotation that was not revealed by IPA pathway analysis but confirmed independently by Reactome annotation in DAVID. Increased transcription of these histone genes may indicate that a greater fraction of cells in the diabetes-exposed embryo are in S-phase, or that cells remain in S-phase for a longer time, thus accumulating increased levels of replication-dependent histone transcripts. Intriguing in this regard is the reversal of correlation to anti-correlation by diabetes exposure and decreased expression of the mouse Timeless gene (Figure 8C), which normally plays a role in stabilizing replication forks (Smith et al., 2009), regulates differentiation and survival of cultured embryonic cells (O'Reilly et al., 2011) and is required for successful implantation (Gotter et al., 2000). Moreover, exposure to diabetes reverses normally anti-correlated to somite-correlated expression of Banp (Birot et al., 2000) (Figure 8D), which associates with BTG3 in an anti-proliferative nuclear complex. The role of Banp in chromatin accessibility and transcriptional activation (Grand et al., 2021) of CpG-island promoters suggests that its increased expression affects a wide variety of transcriptional targets, possibly in many cell types (Schug et al., 2005). This notion is consistent with the IPA pathway annotations presented here, and with our implication of chromatin modifications (Salbaum and Kappen, 2012) and multiple transcriptional programs [(Pavlinkova et al., 2009) and here] in the mouse embryonic response to the exposure to maternal diabetes.

### Absence of a strong transcriptome response by the visceral yolk sac to diabetes exposure

An entirely unexpected result of the present study is that yolk sac samples did not reveal major transcriptome changes upon exposure to maternal diabetes. We detected only 98 genes differentially expressed (at moderate fold-changes), but there was, again, greater variability of profiles among the diabetes-exposed sample when compared to yolk sacs from normal pregnancies. The somite stage of the cognate embryo had some influence, albeit less obvious on the PCA plot for these 98 genes. A microarray analysis of yolk sac yielded a similarly small gene number in diabetic rat pregnancies at later stages (Reece et al., 2006), while species and technical differences, including small sample numbers, could also account for the lack of any congruence to our results. Even among the vast majority of genes not differentially expressed, the influence of somite stage was obvious in the altered distribution of Pearson coefficients in diabetes-exposed samples. Thus, as found for the embryo, exposure of the yolk sac to maternal diabetes uniquely affects the relationship of gene expression levels to developmental maturity. Intriguingly, enriched among genes with a strong correlation were those encoding proteins involved in mitochondrial function and the oxidative phosphorylation pathway, paralleling our findings for embryos. Among the anti-correlated genes, only the micro-RNA biogenesis pathway was shared with the embryo samples.

The near absence of transcriptional response in the yolk sac to diabetes exposure was surprising also in light of multiple reports of yolk sac cellular (Pinter et al., 1986b) and molecular (Pinter et al., 1999) anomalies in diabetic pregnancies, including vasculopathy and reduced hematopoiesis (Nath et al., 2004), although analysis was often performed at later developmental stages. However, lack of differential expression of hemoglobin genes in our yolk sac profiles (data not shown) underscores that blood island formation is not affected by the exposure to maternal diabetes at the somite stages we investigated. In addition, even though we detected significant accumulation of lipid droplets in yolk sacs under diabetic conditions as early as E8.5 (Zhang et al., 2023), this is not reflected in altered transcriptome profiles.

### Implications for understanding molecular mechanisms underlying adverse outcomes in pregnancies complicated by maternal diabetes

In summary, the major conclusions from our study are: 1) Embryo and yolk sac react differently in the exposure to maternal diabetes, with minimal response at the transcriptional level in yolk sac, in contrast to broad changes in multiple pathways in the developing embryo. The implication is that -with the exception of a few genes- transcriptional regulation is governed differently in yolk sac and embryonic cells, likely involving different sets of transcription factors. 2) Developmental stage of the embryo contributes to the increased variation in gene expression profiles upon exposure to maternal diabetes, which -for many genes- has a unique effect of altering the relationship of expression level to somite stage. This has implications for the interpretation of studies with small sample numbers, where equal distribution of somite stages in both normal and experimental groups may not be achieved. 3) For embryos, we identified new pathways as responsive to exposure to maternal diabetes, specifically cholesterol biosynthesis, and NAD metabolism and signaling. Both pathways are amenable to supplementation strategies that can be tested for their potential to prevent of developmental defects in diabetic pregnancies. 4) Additionally, our analyses provide evidence for involvement of large-scale chromatin modifications and altered cell-cycle control, but it is currently unknown whether and to what extent pathways interact in the pathogenesis of birth defects. Implications for prevention or treatment of adverse outcomes of diabetic pregnancies are that pharmacological or nutritional interventions may have to encompass a broad spectrum of targets.

Finally, it should be kept in mind that our investigation was conducted in the NOD mouse strain, which spontaneously develops diabetes that includes immune system involvement also found in human type I diabetes. Similarities to chemically (i.e., Streptozotocin) induced models of diabetes in other mouse strains exist at the cellular level, both related to impaired migration of mesodermal cells (Salbaum et al., 2015) and to excessive lipid accumulation in the visceral yolk sac (Zhang et al., 2023), with commonalities at the transcriptomic level remaining to be determined. Furthermore, we focused on only those conceptuses that contained morphologically normal embryos. Likely, this excluded those individuals that would have manifested neural tube defects later, which we previously showed can be identified at E8.5 by ectopic accumulation of cells protruding from the midline (Salbaum et al., 2015; 2022). Yet, it was only by omitting these morphologically abnormal embryos that we could avoid confounding of outcomes by corollaries of abnormal development. Our results therefore do not immediately imply identification of causes for abnormal development, but reflect the transcriptomic response of embryos and yolk sacs to the intrauterine exposure to maternal diabetes. As this exposure is likely shared by all conceptuses within a given litter, we hypothesize that it raises vulnerability to adverse outcomes overall, and that individual risk for defective development is precipitated by -as of yet unidentified- factors that afflict only a fraction of the individuals. Whether such causal influences originate in the yolk sac, the embryo itself, or arise from their interactions is currently unknown. Investigations of conceptuses with prognostic molecular or morphological features at developmental stages preceding defective neural tube closure will be required to discover the potentially causative or pathogenesis-predicting molecular factors and cellular pathways.

## Data Availability

The datasets presented in this study can be found in online repositories. The names of the repository/repositories and accession number(s) can be found below: https://www.ncbi.nlm.nih.gov/geo/, GSE197396.
